# A comparative study of sexual functioning, depression, anxiety, self-esteem, well-being and close relationships among individuals with and without Diabetes Mellitus

**DOI:** 10.1192/j.eurpsy.2023.1098

**Published:** 2023-07-19

**Authors:** V. Efstathiou, ?. Gjika, K. Gkikas, E. Kaloudi, P. Bali, K. Papazachos, A. Papadopoulou

**Affiliations:** 1 Psychology Department, National and Kapodistrian University of Athens; 2 Second Department of Psychiatry, National and Kapodistrian University of Athens, “Attikon” University General Hospital, Athens, Greece

## Abstract

**Introduction:**

Diabetes mellitus is a chronic progressive disease, which has been associated with various mental and physical health problems, including sexual disorders. However, especially among female patients the potential effects of diabetes on sexual functioning have been understudied.

**Objectives:**

The aim of this study was to investigate the perceived sexual functioning in patients with diabetes mellitus compared to a group of healthy controls, as well as to explore its possible association with depression, anxiety, self-esteem, well-being and adult romantic attachement.

**Methods:**

The study included 125 patients with diabetes and an equal number of healthy controls. All participants completed the following psychometric scales: Experiences in Close Relationships- Revised (ECR-R), Hospital Anxiety and Depression Scale (HADS), Rosenberg Self-Esteem Scale (RSE), Mental Health Continuum Short Form (MHC-SF), as well as Female Sexual Function Index (FSFI) and International Index of Erectile Function (IIEF) for female and male participants, respectively.

**Results:**

The results did not reveal a significant relationship between diabetes and sexual functioning, as no statistically significant differences emerged between patients and healthy controls neither among men nor women. However, in patients with diabetes, a positive correlation was found between perceived sexual dysfunction and depression, anxiety, and avoidant and anxious attachment, as well as a negative correlation with self-esteem and well-being.

**Conclusions:**

The findings highlight the importance of investigating sexual functioning among individuals with diabetes mellitus, especially women, as well as its relationship with crucial psychological factors.

**Disclosure of Interest:**

None Declared

